# More than Just Type 1 or Type 2: Radiologically and Anatomically Refined Lunate Classification Correlating Ulnar Carpal Alignment and Hamate-Lunate Osteoarthrosis

**DOI:** 10.3390/jfmk10020141

**Published:** 2025-04-23

**Authors:** Wolfram Demmer, Lia K. Fialka, Jens Waschke, Irene Mesas Aranda, Elisabeth Haas-Lützenberger, Riccardo Giunta, Paul Reidler

**Affiliations:** 1 Department of Hand, Plastic and Aesthetic Surgery, LMU Klinikum, Ziemssenstraße 5, 80336 Munich, Germany; 2 Chair of Vegetative Anatomy, Institute of Anatomy, Faculty of Medicine, LMU Munich, 80336 Munich, Germany; 3 Department of Radiology, LMU University Hospital, LMU Munich, 81377 Munich, Germany

**Keywords:** lunate type, ulnar-sided wrist pain, hamatolunate osteoarthrosis, hamatolunate impingement, D-line, wrist arthroscopy, hand surgery

## Abstract

**Background:** Hamate-lunate impingement or osteoarthritis can be a cause of ulnar-sided wrist pain. In the literature, the lunate has commonly been classified according to the configuration of its distal articular surface into type 1 and type 2, as described by Viegas. A type 1 lunate possesses only a distal articular surface for the capitate, while a type 2 lunate shows an additional medial facet articulating directly with the hamate. Type 2 lunates have been identified as a risk factor for ulnar-sided wrist pain and the development of osteoarthritis in the midcarpal wrist. However, this does not sufficiently explain all arthritic changes between the hamate and lunate. **Methods:** In this prospective anatomical-radiological cadaver study, 60 wrists were examined. The midcarpal articulation was documented using conventional X-ray, CT arthrography, and anatomical dissection. The study specifically analyzed the positioning of the lunate relative to the hamate apex and its association with the development of hamate-lunate osteoarthritis. For this purpose, the classification by Viegas was refined. Based on posterior-anterior (p.a.) X-ray examinations of the wrist lunates were divided into type 1a, type 1b, and type 2. The type 1a lunate articulates only with the capitate in the midcarpal joint. The type 1b lunate also articulates only with the capitate; however, medially, the apex of the hamate protrudes beyond a Differentiation Line (D-line), which extends from the radial border of the trapezium or the ulnar border of the lunotriquetral (LT) space, without forming a facet with the lunate. A type 2 lunate articulates distally with the capitate and has an additional medial facet with the hamate. **Results:** Osteoarthritis between the hamate and lunate was observed in both Viegas type 1 and type 2 lunates. According to our refined lunate classification, both in situ and radiologically, type 1b and type 2 lunates showed a substantially higher prevalence and severity of hamate-lunate osteoarthritis compared to type 1a lunates. However, there was no significant difference in the prevalence of hamate-lunate osteoarthritis between type 1b and type 2 lunates. **Conclusions:** Assessing lunate type and signs of osteoarthritis is essential when evaluating patients with ulnar-sided wrist pain. Our study demonstrates that osteoarthritis in Viegas type 1 lunate is influenced by the position of the hamate apex relative to the D-line. The refined lunate classification, based on correlated radiological and anatomical studies of the wrist, provides a straightforward method for identifying a potential cause of ulnar-sided wrist pain on p.a. X-rays. This classification can help guide further diagnostic and therapeutic decisions, such as wrist arthroscopy with possible resection of the hamate apex.

## 1. Introduction

Chronic wrist pain is a common clinical issue that is receiving increasing attention regarding its diagnosis and treatment [1,2,3]. Pain in the ulnar wrist often leads to significant limitations in patients’ hand function during daily activities. Identifying the causes of ulnar-sided wrist pain can be challenging, as multiple factors may be involved, including injuries to the ulnar carpal bones, ligament tears, osteoarthritis, tendinitis, and nerve compression [4,5].

Among other causes, the configuration of the mediocarpal lunate joint surface can predispose individuals to ulnar-sided wrist pain due to hamate-lunate impingement or osteoarthritis [6,7,8,9]. The pain is particularly expressed when bearing load with supination of the forearm and wrist with ulnar deviation of the hand [10].

The impingement of the lunate and hamate bone can lead to arthrosis of the proximal pole of the hamate, causing ulnar-sided wrist pain [7,8,9]. Thurston and Stanley first described hamate-lunate impingement as a cause for degenerative changes in the hamate-lunate joint [2]. Also, repeated impaction of the distal lunate medial facet and the apex of the hamate, especially through ulnar deviation, may lead to worsening of the subchondral erosions [6,10]. A significant correlation was found between cartilage erosion and the presence of a medial hamate-lunate facet [2]. The lunate bones are classified based on their midcarpal articular surfaces into type 1 and type 2, according to Viegas. A type 1 lunate is defined by its exclusive articulation with the capitate, whereas a type 2 lunate additionally presents a medial facet that articulates with the hamate [3]. In radiological studies, the frequency of type 1 lunate has been reported to be between 42.5% and 46.1%, while the frequency of type 2 lunate ranges from 53.9% to 57.5%. Type 2 lunates showed a significantly higher incidence of cartilage erosion and signs of osteoarthritis [3,11,12].

Radiographically, osteoarthritis is classified according to Kellgren and Lawrence [13,14]. Cartilage damage is classified arthroscopically according to Outerbridge [15,16]. The two classifications differ in their measurement parameters; however, a high correlation between radiological and arthroscopic joint damage has been demonstrated, particularly in advanced stages of osteoarthritis, such as Outerbridge/Kellgren and Lawrence stages 3 and 4 [17,18,19,20]. In clinically apparent hamate-lunate impingement, a hamate-lunate articulation can frequently be observed during arthroscopy; however, only up to 40% of these articulations are evident in radiographs [2]. Small medial facets of the lunate or early arthritic changes between the lunate and hamate cannot always be detected radiologically [2,3]. Direct observation and evaluation of arthrosis and chondromalacia through wrist arthroscopy is, therefore, the diagnostic method of choice [8]. Additionally, the underlying pathology can be treated through arthroscopic resection of the proximal hamate, leading to an improvement in symptoms [2].

This study aims to examine the importance of the configuration of the midcarpal joint surface of the lunate as well as the ulnar carpal alignment in the development of hamate-lunate osteoarthritis. Based on the classification by Viegas, we specifically analyzed the positioning of the ulnar carpal bones, particularly the apex of the hamate, and were able to refine the existing classification. We propose an extension of the original classification into type 1a, type 1b, and type 2 to improve the clinical assessment of hamate-lunate osteoarthritis.

## 2. Materials and Methods

### 2.1. Study Design and Data Collection

For this prospective anatomical and radiological study, 60 cadaveric wrists from the Anatomical Institute of the Ludwig-Maximilians-University, Munich, were examined. They originated from 30 body donors of the annual dissection course for medical students. Formalin was used to preserve the anatomical specimens [21]. Amputations were conducted at the distal third of the forearm on a total of 30 paired left and right forearms and hands.

The wrists were analyzed using three different modalities. Standardized X-rays and arthrographic Computed Tomography (CT) of the wrists were performed. Subsequently, dissection and in situ documentation of findings were carried out by trained hand surgeons. The data were pseudonymized, and the evaluation of the radiological data was performed in a blinded manner by radiologists from the Clinic of Radiology at LMU Munich. The assessment of the in situ data was carried out by trained hand surgeons from the Department of Plastic, Aesthetic, and Hand Surgery at LMU Munich. The radiological and anatomical datasets were later compared with each other. The Ethics Committee of the Medical Faculty of LMU Munich, Germany, approved the study (approval number: 22-0674, Date 26 August 2022, Amendment 15 June 2023).

### 2.2. Radiological Examinations

The detached wrists were examined in pairs. Posterior-anterior (p.a.) and lateral radiographs were performed, as well as radial and ulnar stress views [22]. The X-ray examination was conducted with a Ysio Max radiography system by Siemens Health Engineers (Forchheim, Germany).

The degree of osteoarthritis (osteoarthrosis) between the lunate and hamate was determined according to the Kellgren-Lawrence classification of osteoarthritis (Table 1) [13,14,23].

**Figure 1:**
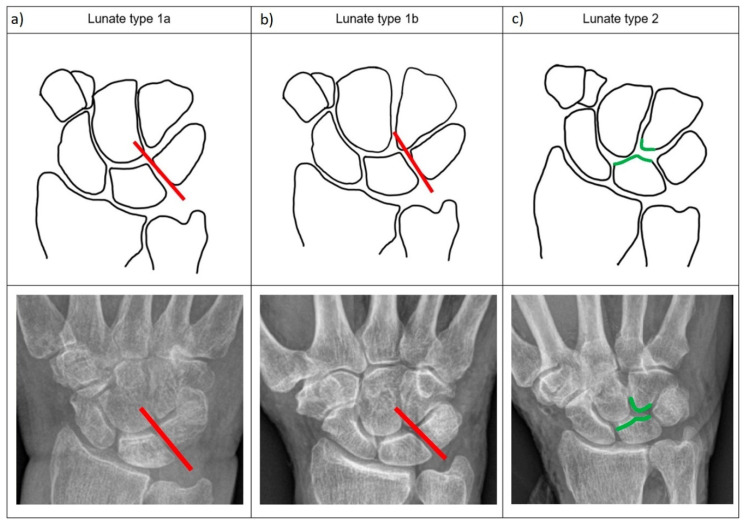
Refined lunate classification based on the configuration of the mediocarpal joint surface of the lunate, as well as the position of the apex of the os hamatum in relation to the ulnar edge of the LT space (red line) in standardized p.a. radiographs: (**a**) Type 1a lunate, with the hamate apex ulnarly located relative to the D-line; (**b**) Type 1b lunate, with the hamate apex positioned radially to the D-line; (**c**) Type 2 lunate presenting a medial hamatolunate joint (green), as described by Viegas [3].

The presence of a distal medial facet on the joint surface of the os lunatum in the p.a. view was documented, and the lunate was classified according to Viegas classification as type 1 or type 2 [3]. Further differentiation of type 1 lunates was performed according to the alignment of the ulnar carpal bones. The position of the apex of the os hamatum was determined relative to the D-line, extending distally to the radial edge of the articular surface of the triquetrum or the ulnar border of the LT space, respectively, in p.a. radiographs. Type 1 lunates were subclassified into type 1a, where the apex of the os hamatum does not exceed the D-Line radially, and type 1b, where the apex of the os hamatum exceeds this line (Figure 1). To correctly interpret the crossing of the D-line by the apex of the hamate, a strictly standardized PA X-ray of the wrist is necessary.

All 60 hands also received CT arthrography to evaluate the type of lunate and the degree of hamatolunate osteoarthrosis. As a contrast agent, Imeron 300 was diluted to 50% with NaCl and injected into the mediocarpal, radiocarpal, and distal radioulnar joints of all specimens [24,25]. The CT arthrographies were obtained using the Revolution CT 256 system (GE HealthCare Technologies Inc., Chicago, IL, USA), with the tomographic thickness range being selected from 0.63 mm to 1.5 mm.

The localization and degree of osteoarthrosis in the radiocarpal and midcarpal joints were graded and documented, applying the Kellgren-Lawrence classification for radiographs in the coronal CT plane [14,26,27,28]. In addition, the type of the lunate bone, according to Viegas, and the relative position of the apex of the os hamatum, as described above, were noted.

### 2.3. Anatomical Examinations

After the radiological examination, the wrists were dissected and evaluated by trained hand surgeons. All wrists were intact and closed before the examination. The dissection of the wrist was performed through a universal dorsal approach. A T-shaped incision was made to open the wrist capsule [29]. The radiocarpal and mediocarpal joints were inspected and subsequently separated from each other. Osteoarthritis in the hamatolunate joint was documented according to the Outerbridge classification (Table 2) [15,16]. A detailed photographic documentation of the findings was carried out.

### 2.4. Statistical Analysis

The results were statistically analyzed using SPSS (version 29; IBM Corp., Armonk, NY, USA), with a significance level set at *p* ≤ 0.05 for all tests, including Shapiro-Wilk, Chi-Square, Mann-Whitney, and Kruskal-Wallis. Spearman’s rank correlation coefficient (ρ) and Cramer’s V were determined to assess correlations between groups. The size of our study population was verified in advance and confirmed for significant results (Cohen’s d = 0.8, alpha level 0.05, power 0.8) [30,31].

## 3. Results

**Figure 2:**
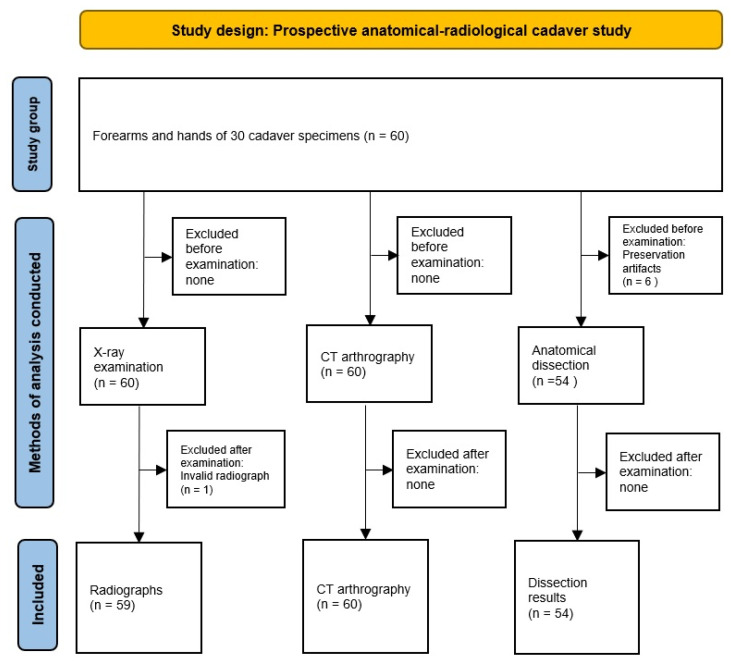
Study design. Sixty hands from thirty cadavers were examined using X-ray, CT arthrography, and anatomical dissection.

Of the 30 body donors, 42 female and 18 male cadavers, with 60 wrists, were included in our study. The age of the donors ranged from 57 to 93 years, with a mean age of 81.13 (SD ± 9.63). A total of 59 wrists were examined radiologically, 60 wrists were assessed using CT arthrography, and 54 wrists were evaluated anatomically in situ (Figure 2).

One radiograph had to be excluded from the radiological evaluation for technical reasons. In the anatomical in situ examination, 6 wrists were excluded due to significant preservation artifacts that made evaluation impossible.

In all examination methods applied, age, gender, and the side of the hand showed no correlation with the type of lunate according to the Viegas Classification or the prevalence of osteoarthritis.

Initially, the mediocarpal joint surface of the lunate bone was classified into type 1 and type 2, according to Viegas [3]. In the X-ray examination, 74.6% (n = 44) were classified as type 1 and 25.4% (n = 15) as type 2. In the CT arthrography, 71.7% (n = 43) were classified as type 1 and 28.3% (n = 17) as type 2. The direct assessment of the anatomical in situ data revealed 42.6% (n = 23) type 1 and 57.4% (n = 31) type 2 lunates (Table 3). In the radiological examinations, no significant frequency difference was found between lunate type 1 and type 2 in X-ray or CT arthrography. However, type 2 lunates were seen more frequently in the in situ data compared to X-ray and CT arthrography.

**Figure 3:**
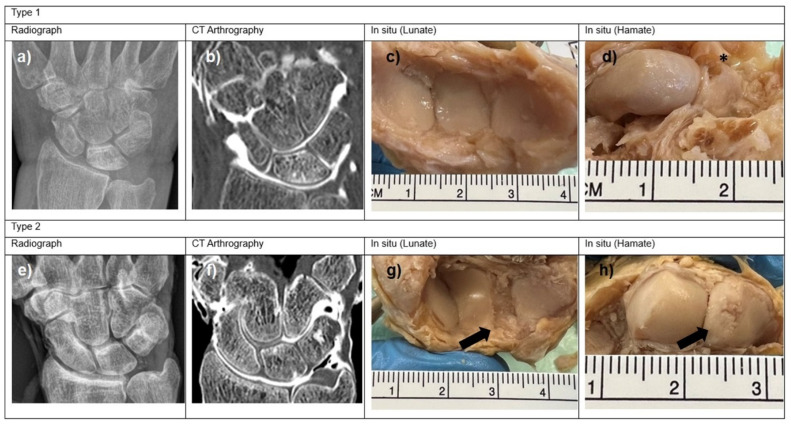
Radiographs, CT arthrographs, and in situ photographic documentation of lunate type 1 (**a**–**d**) and lunate type 2 (**e**–**h**) conducted on the same specimens. Arrows indicate osteoarthritis, and Asterix indicates preparation artifact (fracture).

The hamatolunate joint was examined for osteoarthrosis using radiographs, CT arthrographies, and direct anatomical examination in situ (Figure 3).

In situ:

Of the 23 lunates anatomically rated as type 1, 14 (60.9%) showed signs of arthrosis, such as cartilage damage and osteoarthritis, while 9 (39.1%) showed no signs of arthrosis. Of those type 1 lunates presenting hamatolunate arthrosis, 4 (17.4%) showed grade 2, 9 (39.1%) grade 3, and 1 (4.3%) grade 4 osteoarthritis according to the Outerbridge grading scale. Of the 31 anatomically classified type 2 lunates, 27 (87.1%) presented with osteoarthritis, while only 4 (12.9%) showed no signs of arthrosis. Among the specimens with signs of arthrosis, 5 (16.1%) showed grade 2 osteoarthritis, 15 (48.4%) grade 3 osteoarthritis, and 7 (22.6%) grade 4 arthrosis according to the Outerbridge scale (Table 4) [15,16].

Radiological imaging:

X-ray examinations showed hamatolunate osteoarthritis in 81.4% (n = 59) of all specimens. When differentiating lunates according to Viegas classification, only 33 (75.0%) of the 44 types 1 lunates showed radiographical signs of arthrosis. Whereas in all of the 15 type 2 lunates (100%), radiological signs of osteoarthritis were detected (Table 2). Next to the expected frequent occurrence of osteoarthritis in type 2 lunates, the remarkably high rate of radiologically detectable hamatolunate osteoarthritis in type 1 lunates prompted further investigation into its cause.

Taking into account the alignment of the ulnocarpal bones, type 1 lunates were divided into two subtypes based on the location of the tip of the hamate bone and the LT Space in p.a. radiographs, as described in the methods section. Three types of lunates were thus classified: Type 1a, type 1b, and type 2 (Figure 1).

A type 1a lunate articulates distally only with the capitate. It has no contact with the hamate and the articular surface of the triquetrum exceeds the hamate. A type 1b lunate articulates distally also only with the capitate and does not have an additional medial facet. However, the radial edge of the articular surface of the triquetrum, or the ulnar border of the LT Space, is exceeded by the apex of the hamate (Figure 1a,b). A type 2 lunate is defined by having an additional medial facet to the hamate and articulating distally with the capitate (Figure 1c).

In the p.a. X-ray pictures (n = 59), 32 (54.2%) lunates were classified type 1a, 12 (20.3%) were classified type 1b, and 15 (25.4%) were classified type 2. In CT arthrographies of the wrists (n = 60), the lunates were classified in 32 (53.3%) type 1a, 11 (18.3%) type 1b, and 17 (28.3%) type 2. In X-ray and CT arthrography, the total number of lunate types corresponded to each other (Cramer V: 0.964). The degree of hamatolunate osteoarthritis was determined in X-rays and CT arthrography using the Kellgren-Lawrence score. The osteoarthritis score of X-ray and CT arthrography results correlated strongly (ρ = 0.828, *p* < 0.001).

X-ray examination showed signs of arthrosis in 65.6% (n = 21) of the type 1a lunates and in 100% of both type 1b (n = 12) and type 2 lunates (n = 15).

Of the type 1a lunates presenting signs of hamatolunate arthrosis, 6 (18.8%) were classified as grade 1 arthrosis, 14 (43.8%) were classified as grade 2, and 1 (3.1%) was classified as grade 3 arthrosis. Arthrosis in the 12 type 1b lunates was rated as grade 1 in 2 (16.7%) cases, grade 2 in 7 (58.3%), grade 3 in 2 (16.7%), and grade 4 in 1 (8.3%) case. Of the 15 type 2 lunates, 9 (60.0%) presented grade 2 arthrosis, 4 (26.7%) grade 3 arthrosis, and 2 (13.3%) grade 4 arthrosis (Table 4).

In CT arthrographies, 65.6% (n = 21) of the type 1a lunates showed signs of arthrosis. Again, 100% of both the type 1b and type 2 lunates showed signs of arthrosis. Of the type 1a lunates presenting hamatolunate arthrosis, 10 (31.3%) had grade 1 arthrosis, 10 (31.3%) had grade 2, and 1 (3.1%) had grade 3 arthrosis. In type 1b lunates, 2 (18.2%) were rated as grade 1 arthrosis, 6 (54.5%) were classified as grade 2, 2 (18.2%) were classified as grade 3, and 1 (9.1%) was classified as grade 4 arthrosis. The 17 type 2 lunates were divided into 11 (64.7%) with grade 2 arthrosis, 4 (23.5%) with grade 3, and 2 (11.8%) with grade 4 arthrosis (Table 4).

Radiographic classification of lunates into types 1a, 1b, and 2 served as the basis for further investigations. According to their classification on p.a. X-rays, the individual lunates were correlated with the in situ presence of osteoarthritis.

**Figure 4:**
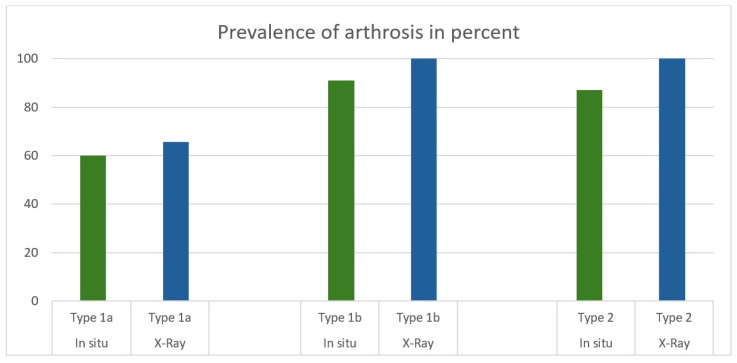
Prevalence of arthrosis in the subclassified lunates in X-ray and in situ, using the radiologic differentiation of type 1a and type 1b for the in situ data.

It was observed that lunates graded as type 1b according to the refined classification exhibited a substantially higher prevalence of hamatolunate osteoarthritis (90.9%) in situ compared to type 1a lunates (60.0%). Similarly, a significant difference was identified between the in situ presence of osteoarthritis when comparing type 1a lunates to type 2 lunates. On the other hand, no significant difference was found regarding the in situ presence of osteoarthritis between type 1b lunates and type 2 lunates (Figure 4).

**Figure 5:**
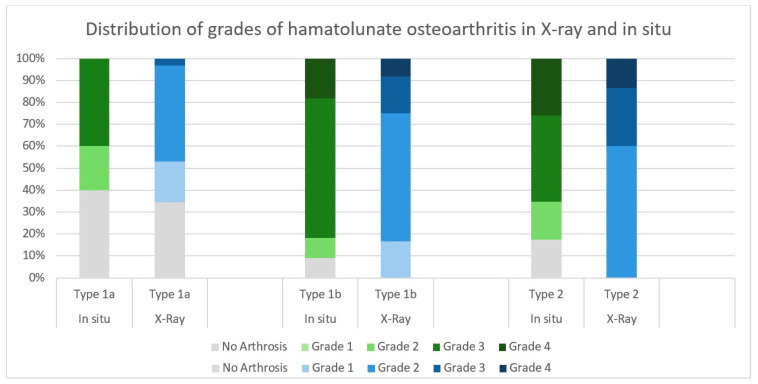
Degree of hamatolunate osteoarthrosis broken down by lunate types. Green bars show the distribution in X-ray analysis (Kellgren-Lawrence classification), while blue bars represent the distribution of severity grades of chondral damage in the in situ examination (Outerbridge classification) [14,15].

When comparing the degree of osteoarthritis between type 1a and type 1b lunates, a significant difference was evident radiologically as well as in situ. Type 1a lunates had an average osteoarthritis grade of 1.16 in radiographs [14] and 1.6 in situ [15], while type 1b lunates presented a significantly higher mean degree of 2.17 [14] in Radiographs and 2.8 in situ [15]. The previously radiologically subclassified lunates were analyzed for visible arthrosis between the hamate and lunate and classified according to the Outerbridge system. Type 1a lunates showed an average visible grade of arthrosis of 1.6, while type 1b showed an average of 2.8. The difference was significant. The distribution of the severity grades of arthrosis in X-rays, recorded according to Kellgren-Lawrence, and the in situ grades of arthrosis according to Outerbridge had a positive Spearman’s correlation (ρ = 0.442; *p* < 0.001) (Figure 5).

In order to rule out other potential biasing factors for osteoarthritis, the wrists were screened radiologically and in situ for lesions of intracarpal ligaments and consecutive signs of SLAC (scapholunate advanced collapse) and SNAC (scaphoid non-union advanced collapse) wrist. No scaphoid non-union, and thus no SNAC wrist, was found in our study population. One SLAC 4 wrist was identified in a type 1b lunate, and two SLAC 3 wrists were observed without arthrosis of the capitolunate facet of the lunate [32,33].

LT ruptures were more common, with an LT lesion found in half of the wrists (n = 27). However, the distribution incidence among lunate types 1a, 1b, and 2 was not significant (*p* = 0.372).

## 4. Discussion

Ulnar-sided wrist pain is a common cause of upper extremity disability, potentially impacting the daily activities of the patient and the overall function of the hand [5,34]. Most clinical and radiological tests focus on ulnar impaction and TFCC lesions [35,36]. For the assessment of possible other less common causes of ulnar-sided wrist pain, such as hamatolunate impingement or subsequent hamatolunate osteoarthritis, radiological criteria are scarcely described. The presence of a medial facet of the lunate is commonly associated with a higher risk of developing hamatolunate arthrosis [3,11,12,37]. Other guidelines to assess possible hamatolunate impingement or osteoarthritis in regular X-ray examinations have not yet been described.

The setup of this research as a combination of an anatomical and radiological study provided the opportunity to evaluate the ulnar carpus using two radiological techniques, X-ray and CT arthrography, as well as in situ inspection of the same specimens. This enabled a particularly valuable correlation between the radiological and anatomical arthrotic changes in the hamatolunate joint.

When analyzing the radiological data and classifying the lunate according to the commonly used Viegas classification, a notably higher number of type 1 lunates was found. The data showed a prevalence of 74.6% and 71.7% for type 1 and 25.4% and 28.3% for type 2 in X-ray and CT arthrography, respectively. Also, considerably more X-ray-classified type 1 lunate exhibited osteoarthritis than suggested in the relevant literature [3,11,12]. The distribution of lunate types has been reported between 29–46.1% for type 1 and 53.9–71% for type 2 [3,11,12]. It should be noted that purely anatomical studies describe type 1 lunates considerably less frequently and type 2 more frequently [12].

Consistent with this, we observed a significantly higher number of type 2 lunates was observed in the anatomical in situ examinations of the same wrist, with 42.6% type 1 and 57.4% type 2. Radiological detection of a small medial facet of the lunate and, thus, an articulation between the hamate and lunate bones is rather difficult and cannot always be confirmed [3]. Thurston and Stanley described that only 40% of their articulations studied by arthroscopy were radiologically detectable [2]. Our study confirms the literature in that the frequency of articular contact between the hamate and lunate appears to be tendentially underestimated when examined radiologically.

Previously, type 2 lunates have been primarily described as a predisposing factor for hamatolunate osteoarthritis [3,11,12,37]. Accordingly, a prevalence of osteoarthritis in wrists presenting a type 2 lunate was found both radiographically and in situ. Furthermore, the rate of osteoarthritis in type 1 lunates was noticeably higher than expected. When assessing type 1 lunates radiographically and in situ, signs of osteoarthritis were observed in 75% and 69.9%, respectively. Further analysis revealed a correlation between the occurrence of osteoarthritis and the position of the hamate apex relative to the radial edge of the triquetrum’s articular surface. A line (D-line) extending from the radial border of the trapezium was established to subclassify type 1 lunates. A protrusion of the hamate apex radial to this line was identified as a predisposing factor for hamatolunate osteoarthritis.

Based on the classification by Viegas, we defined type 1a and type 1b lunates according to the apex of the hamate relative to the D-line in p.a. X-rays with 0° rotation [5]. Special care must be taken to ensure this standardized view in order to correctly interpret the lunate types. Ulnar and radial deviation of the wrist reduce the diagnostic value of the examination. Both type 1a and type 1b lunates show only one articulating surface with the capitate. In type 1b lunates, the apex of the hamate protrudes more radially, crossing the D-line radially. In type 1a lunates, the apex remains ulnar to this line. After reclassifying all type 1 lunates in p.a. X-rays, we compared the occurrence and severity of hamatolunate osteoarthritis across all three modalities: radiography, CT arthrography, and in situ.

In radiological examinations, including X-ray and CT arthrography, a high correlation was found between both methods when classifying lunates into type 1a, type 1b, and type 2 (Cramer V: 0.964). This suggests that X-ray is a sufficiently specific radiological method to differentiate lunate type 1a and type 1b for clinical use. Furthermore, a high correlation in the severity of hamatolunate osteoarthritis could be detected when grading type 1a, type 1b, and type 2 lunates Furthermore, a high correlation in the severity of hamatolunate osteoarthritis was observed when grading type 1a, type 1b, and type 2 lunates (ρ = 0.828, *p* < 0.001), thus X-ray also represents a sensitive method for detecting and classifying manifest hamatolunate arthrosis [38,39] (Table 3).

The in situ analysis of signs of hamatolunate osteoarthritis in previously radiologically pre-classified lunates (type 1a and type 1b) showed a positive correlation between osteoarthritic changes diagnosed on p.a. radiographs and the hamatolunate osteoarthritis observed in situ. When comparing the degree of osteoarthrosis, the radiographic staging into severity grades 1–4, according to Kellgren-Lawrence, showed a positive correlation with the in situ-determined severity grades 1–4 according to Outerbridge (ρ = 0.442; *p* < 0.001) (Table 3). In the literature, the comparative application of the Kellgren-Lawrence score and the Outerbridge classification is described when comparing clinical-anatomical and radiological findings [18,40].

Our study confirms the comparability of the two classifications in the context of the carpal region. In both the radiological and in situ examinations, type 1b lunates showed considerably more frequent and more severe signs of hamatolunate osteoarthritis than type 1a lunates. The occurrence of arthrosis was 100% in X-rays and 90.9% in situ for type 1b lunates, whereas type 1a lunates showed a prevalence of 65.6% in X-rays and 60% in situ. Radiologically, there was a significant difference in the degree of hamatolunate osteoarthritis between type 1a and type 1b in p.a. X-rays, with type 1b showing more severe damage (*p* = 0.02). A significant difference in the occurrence and degree of osteoarthritis was demonstrated between type 1a and type 2 lunates (*p* = 0.01), with type 2 showing considerably more severe osteoarthritic changes. On the other hand, there was no significant difference in the occurrence or severity of hamatolunate osteoarthritis between type 1b and type 2 lunates. The data strongly supports our hypothesis that type 1b, such as type 2, predisposes individuals to the occurrence and severity of hamatolunate osteoarthritis.

In assessing differential diagnoses of ulnar wrist pain with regard to a possible hamatolunate impingement or early osteoarthritis, our refined lunate classification provides a reliable, specific, and sensitive tool, requiring only a simple p.a. X-ray of the wrist. To our knowledge, no other method for assessing hamatolunate osteoarthritis risk refers to the configuration of the ulnar carpus. Other previously described extensions of the lunate classification also mention an intermediate lunate type [41,42]. However, it is solely described in regard to the kinematic aspects of wrist motion and not in correlation with osteoarthritic changes. For comparison, we also correlated this intermediate lunate, defined by a distance of >2 mm and <4 mm between the os capitatum and os triquetrum on p.a. X-rays, with osteoarthritic occurrences both radiologically and in situ. The correlation was not significant (*p* = 0.051).

Other factors, such as SLAC or SNAC wrist, could largely be ruled out as confounding variables. LT ruptures were common; however, their distribution among the defined groups of lunate types 1a, 1b, and 2 was not significant. LT ruptures may contribute to hamate arthrosis as part of the hamate arthrosis lunotriquetral ligament tear (HALT) process [43]. However, within the scope of this study, no conclusions can be drawn regarding the role of the LT ligament in the development of hamatolunate osteoarthritis.

Impingement of the lunate and hamate can lead to arthrosis of hamtolunate joint, causing ulnar-sided wrist pain [2,7,9]. In addition to the generally described medial joint facet of the distal lunate, we were able to identify the configuration of the ulnar wrist, particularly the apex of the hamate in relation to the D-line, as a decisive predisposing factor. The refined lunate classification presented here allows for the assessment of this risk with a simple p.a. X-ray of the hand. In vivo, verification of the findings and potential therapy through arthroscopy is recommended as the gold standard for the treatment of hamatolunate impingement and osteoarthritis.

Limitations of this study:

The use of anatomical specimens enabled a multimodal examination with X-ray, CT arthrography, and in situ inspection. However, the fixation of the specimens in formalin can lead to artifacts such as demineralization of the bone, altering the mechanical properties of the tissue [21,44]. The cancellous architecture of the subchondral bone, along with any potential degenerative changes, remains largely unchanged [45]. During the fixation process, cartilage dehydrates. At the same time, collagen fibers become densely packed and hardened, making it difficult to detect early stages of chondral damage such as Outerbridge stages 1 and 2 [44,46]. Also, the study population consisted of elderly body donors (average age 81.13 (SD ± 9.63) years), and the sample size was limited. To validate the results, they should be cross-checked under clinical in vivo conditions with a larger study population. Patients undergoing diagnostic wrist arthroscopy due to ulnar-sided wrist pain should be preoperatively assessed in p.a. wrist X-rays using the described modified lunate classification to evaluate the risk of osteoarthritis and then compare it with in vivo arthroscopic findings [8,43,47].

## 5. Conclusions

Hamatolunate osteoarthrosis can be a cause of ulnar-sided wrist pain. The prevalence of osteoarthritis is significantly increased in type 2 lunates but also in cases where the apex of the hamate extends radially beyond the D-line, an extension of the radial border of the trapezium. In such cases, contact between the hamate and lunate is likely, even without a radiologically visible medial lunate joint facet.

We have, therefore, refined the existing lunate classification and now differentiate between type 1a, type 1b, and type 2 lunates based on p.a. radiographs. This distinction allows for easy identification of the predisposition for hamatolunate impingement or osteoarthritis as a possible differential diagnosis in a clinical setting. This, in turn, facilitates decision-making for further treatment, such as wrist arthroscopy with possible resection of the hamate apex.

## Figures and Tables

**Table 1:** Kellgren-Lawrence classification for radiological grading of osteoarthritis [14].

Grade of Osteoarthritis	Radiographic Evidence of Osteoarthritis
0	none
1	doubtful
2	minimal
3	moderate
4	severe

**Table 2:** Outerbridge classification for the arthroscopic grading of osteoarthritis [15,16].

Grade of Osteoarthritis	Surface Description of Articular Cartilage and Diameter of Lesion
0	normal cartilage
1	chondral lesions characterized by softening and swelling
2	partial-thickness defect with fissures not exceeding 1.3 cm in diameter and not reaching the subchondral bone
3	Fissures of the cartilage >1.3 cm in diameter reaching the subchondral bone
4	erosion of the articular cartilage that exposes subchondral bone

**Table 3:** The distribution of lunate types according to the Viegas classification was analyzed across the examination modalities of X-ray, CT arthrography, and in situ inspection [3].

	Type 1	Type 2
X-ray	44 (74.6%)	15 (25.4%)
CT arthrography	43 (71.7%)	17 (28.3%)
In situ	23 (42.6%)	31 (57.4%)

**Table 4:** Classification of lunates into their subtypes with the general prevalence of arthrosis and the respective arthrosis grades (^1^ Graded according to Outerbridge classification system, ^2^ Graded according to Kellgren-Lawrence-Score).

	Luntate Type	Total	Overall Percentage of Osteoarthritis	Arthrosis Grade			
				1	2	3	4
In situ	1	42.6% (n = 23)	60.9%	0.0% (n = 0) ^1^	17.4% (n = 4) ^1^	39.1% (n = 9) ^1^	4.3% (n = 1) ^1^
	2	57.4% (n = 31)	87.1%	0.0% (n = 0) ^1^	16.1% (n = 5) ^1^	48.4% (n = 15) ^1^	22.6% (n = 7) ^1^
X-Ray	1a	54.3% (n = 32)	65.6%	18.8% (n = 6) ^2^	43.8% (n = 14) ^2^	3.1% (n = 1) ^2^	0% (n = 0) ^2^
	1b	20.3% (n = 12)	100%	16.7% (n = 2) ^2^	58.3% (n = 7) ^2^	16.7% (n = 2) ^2^	8.3% (n = 1) ^2^
	2	25.4% (n = 15)	100%	0% (n = 0) ^2^	60% (n = 9) ^2^	26.7% (n = 4) ^2^	13.3% (n = 2) ^2^
CT	1a	53.3% (n = 32)	65.6%	31.3% (n = 10) ^2^	31.3% (n = 10) ^2^	3.1% (n = 1) ^2^	0.0% (n = 0) ^2^
	1b	18.3% (n = 11)	100%	18.2% (n = 2) ^2^	54.5% (n = 6) ^2^	18.2% (n = 2) ^2^	9.1% (n = 1) ^2^
	2	28.3% (n = 17)	100%	0.0% (n = 0) ^2^	64.7% (n = 11) ^2^	23.5% (n = 4) ^2^	11.8% (n = 2) ^2^

## Data Availability

The original data presented in the study are openly available in PubMed at DOI.
